# The association of gene polymorphisms of adenosine and dopamine receptors with the response to caffeine citrate treatment in infants with apnea of prematurity: a prospective nested case-control study

**DOI:** 10.1186/s13052-024-01776-w

**Published:** 2024-10-29

**Authors:** Jiangbiao Xie, Wei Zhuang, Yao Zhu, Zhi Zheng, Yanru Huang, Simin Ma, Xinzhu Lin

**Affiliations:** 1 Department of Neonatology, Women and Children’s Hospital, School of Medicine, Xiamen University, Xiamen, 361003 Fujian China; 2 Xiamen Key Laboratory of Perinatal-Neonatal Infection, Xiamen, China; 3 Department of Pharmacy, Women and Children’s Hospital, School of Medicine, Xiamen University, Xiamen, China; 4 Department of Central Laboratory, Women and Children’s Hospital, School of Medicine, Xiamen University, Xiamen, China

**Keywords:** Apnea of prematurity, Caffeine, Adenosine receptor, Dopamine receptor, Polymorphism, Nomogram

## Abstract

**Background:**

To investigate the potential influence of adenosine and dopamine receptor genes polymorphisms in combination with clinical factors on the response of preterm infants to caffeine citrate treatment in apnea of prematurity (AOP).

**Methods:**

A prospective nested case-control study enrolled 221 preterm infants with gestational age < 34 weeks. These infants were divided into the response (*n* = 160) and the non-response groups (*n* = 61). 22 single-nucleotide polymorphisms in adenosine and dopamine receptor genes were genotyped. The basic characteristics and clinical outcomes of the two groups were compared. Univariate logistic regression analysis was performed to evaluate the differences in genotype distribution between the groups. Multivariable logistic regression analysis was performed to identify independent risk and protective factors and develop a nomogram to predict caffeine citrate response in preterm infants.

**Results:**

Preterm infants in the non-response group had lower gestational age, lower birth weight, longer periods of oxygen supplementation and caffeine citrate use, and higher incidence of patent ductus arteriosus (PDA), bronchopulmonary dysplasia (BPD), neonatal respiratory distress syndrome (NRDS), retinopathy of prematurity (ROP), and brain injury (*P* < 0.05 for all). The *ADORA1* rs10920573, *ADORA2B* rs2015353, *ADORA3* rs10776728, *DRD3* rs7625282, and *DRD3* rs6280 gene polymorphisms were associated with caffeine citrate response in preterm infants (*P*_*FDR*_ < 0.05 for all). The *ADORA1* rs10920573 CC (aOR, 3.51; 95% CI, 1.34–9.25) and *DRD3* rs6280 CT genotypes (aOR, 3.19; 95% CI, 1.53–6.65) were independent risk factors for non-response, whereas greater gestational age (aOR, 0.631; 95% CI, 0.53–0.75) was an independent protective factor for response. The concordance index of the nomogram was 0.764 (95% CI, 0.687–0.842), and the calibration and decision curve analysis indicated the nomogram had excellent predict performance.

**Conclusions:**

Adenosine receptor gene and dopamine receptor gene polymorphisms influence caffeine citrate treatment response in AOP. By combining genetic and clinical variables, it is possible to predict the response to caffeine citrate treatment in preterm infants.

**Supplementary Information:**

The online version contains supplementary material available at 10.1186/s13052-024-01776-w.

## Background

Apnea of prematurity (AOP) is among the most common diseases associated with premature birth, and its incidence is negatively correlated with gestational age at birth [1]. Frequent episodes of AOP lead to persistent hypoxemia events in preterm infants, and these subjects are exposed to the possibility of prolonged mechanical ventilation, with an increased risk of respiratory failure, cardiovascular problems, and intracranial hemorrhage [2].

Caffeine, as a methylxanthine drug, can effectively stimulate the respiratory center. It has now become the preferred medication for the prevention and treatment of AOP. The Caffeine Therapy for Apnea of Prematurity (CAP) trial confirmed that the use of caffeine not only effectively reduced the occurrence of AOP but also reduced the risk of bronchopulmonary dysplasia (BPD) and improved short- and long-term neurocognitive functions [3–5]. Although caffeine citrate can provide both short- and long-term benefits in preterm infants, its efficacy varies widely in preterm infants [6]. The reasons for individual variations in response are not yet fully understood; however, emerging research suggests that such variations are closely related to genetic factors [7].

As an adenosine analog, caffeine acts by blocking adenosine receptors to generate respiratory stimulation. In addition, it activates ryanodine receptor 2 (RyR2) channels and non-selectively inhibits phosphodiesterases (PDEs), which improve neuromuscular function [8]. The therapeutic effect of caffeine citrate in AOP mainly relies on adenosine receptor blockade. Recent researches have reported that adenosine receptor gene polymorphisms affect preterm infants’ response to caffeine citrate [9, 10]. Adenosine receptors are widespread in the brain and interact with multiple neurotransmitters. In particular, dopamine receptors are colocalized and interact functionally with adenosine receptors. Dopamine receptor gene polymorphisms have been reported to influence individual response to caffeine [11]. However, to our knowledge, the specific effects of these gene polymorphisms on preterm infants’ response to caffeine citrate have not been determined. We speculate that dopamine receptor gene polymorphisms influence the response of preterm infants to caffeine citrate.

Our research focused on investigating the potential impact of dopamine receptor gene polymorphisms on the response of preterm infants to caffeine citrate. We also aimed to identify predictive biomarkers for non-response to caffeine citrate and established a predictive model. The findings provided valuable evidence for the clinical use of caffeine citrate in infants with AOP and facilitated personalized medication adjustments in preterm infants.

## Methods

### Study population

The present study was a prospective nested case-control study conducted at the Neonatal Intensive Care Unit (NICU) of Women and Children’s Hospital, School of Medicine, Xiamen University from October 2021 to June 2023. The study was registered with the Chinese clinical trial registry (http://www.chictr.org.cn/), registration number: ChiCTR2100050212, registration data: 22 August 2021.The study adhered to the tenets of the Helsinki Declaration and was approved by the Ethics Committee of the Women and Children’s Hospital, School of Medicine, Xiamen University (No. KY-2020-040).

The inclusion criteria were as follows: Infants who were born at a gestational age of < 34 weeks and received standard-dose caffeine citrate within 24 h after birth for preventing or treating AOP.

The exclusion criteria were as follows: (I) early-onset sepsis (EOS), (II) grade III–IV intraventricular hemorrhage, (III) severe congenital malformations affecting respiration, (IV) congenital cyanotic heart disease, (V) genetic diseases or chromosomal anomalies, (VI) infants with unqualified umbilical artery blood sampling DNA concentration, and (VII) infants whose treatment was interrupted due to parental wishes based on financial constraints, leading to automatic discharge of the patient.

### Data collection and definitions

The following information was collected: (1) maternal age and pregnancy-related diseases (gestational diabetes and gestational hypertension), (2) basic characteristics of preterm infants [gestational age, birth weight, small for gestational age (SGA), completed antenatal steroids, 5-min Apgar score, neonatal respiratory distress syndrome (NRDS), and patent ductus arteriosus (PDA)], (3) respiratory treatment information [duration of noninvasive ventilation (NIV), invasive mechanical ventilation (IMV) use, duration of IMV, and re-intubations after extubation], (4) caffeine citrate treatment-related information (average maintenance dose and duration of caffeine citrate use), and (5) complications [BPD, necrotizing enterocolitis (NEC), retinopathy of prematurity (ROP), brain injury (including I–II intraventricular hemorrhage and periventricular white matter softening), and death].

The following definitions of various parameters applied to the study: (1) AOP: cessation of breathing for 20 s or longer or a shorter pause accompanied by bradycardia (< 100 beats per minute) and/or hypoxia (oxygen saturation < 85%) in preterm infants with a gestational age of < 37 weeks [1]. (2) Completed antenatal corticosteroid therapy: Intramuscular betamethasone cycle in two doses of 12 mg over a 24-h period within 7 days from the delivery. (3) SGA: A birth weight below the 10th percentile for the same sex and gestational age. (4) PDA: Persistence of the ductus arteriosus for more than 72 h after birth as confirmed by echocardiography. (5) BPD: Diagnosed according to the 2001 National Institutes of Health criteria. For preterm infants with a gestational age < 32 weeks, the severity of BPD was assessed at postmenstrual age 36 weeks. For preterm infants with a gestational age ≥ 32 weeks, the severity of BPD was assessed at > 28d but < 56 d. The severity of BPD including (A) mild, breathing room air; (B) moderate, FiO_2_ 21–30%; (C) severe, FiO_2_ ≥ 30% or requiring positive pressure ventilation or mechanical ventilation [12]. (6) NEC: Diagnosed according to the Bell criteria [13]. (7) ROP: Defined according to agreement with international classification [14]. (8) NRDS: Diagnosed according to the Montreux definition [15]. (9) EOS: Defined as a sepsis occurring within 72 h of birth, which includes culture-positive and clinically proven EOS neonates according to the consensus of Chinese experts (2019 version) [16]. (10) Intraventricular hemorrhage (IVH): Diagnosed and classified according to the described by Papile et al. [17].

### Treatment and group

All eligible preterm infants received appropriate respiratory support based on their condition at admission. Within 24 h of admission, a loading dose of 20-mg/kg of caffeine citrate^®^ [1 mL: 20 mg, containing 10 mg of caffeine and 10 mg of citrate (approved by the National Medical Products Administration with registration number H20163401 and manufactured by Chengdu Yuandong Biopharmaceutical Co., Ltd.)] was intravenously administered. Subsequently, a maintenance dose of 5 mg/kg/day was introduced. If preterm infants achieved full enteral feeding (oral intake of 150 mL/kg), intravenous caffeine citrate was switched to oral administration. After correcting the gestational age to 34–35 weeks and no AOP for 5–7 days, caffeine citrate was discontinued. Oxygen saturation and heart rate were continuously monitored using Radical-7 (USA) with alarms set to alert at oxygen saturation < 85% and heart rate < 100 beats per minute. The occurrence of AOP was determined by the attending physician if the infant had cessation of breathing for > 20 s or longer or a shorter pause accompanied by bradycardia (< 100 beats per minute) and/or hypoxia (oxygen saturation < 85%).

Based on the occurrence of AOP after caffeine citrate treatment, preterm infants were divided into non-response and response groups. The non-response group was defined as the occurrence of AOP more than once a day or as the occurrence of a single episode of severe AOP requiring bag and mask ventilation with supplemental oxygen within 3 days of using caffeine citrate. Non-response preterm infants were temporarily given an additional dosage of 5–10 mg/kg of caffeine citrate, and the daily maintenance dose was increased from 5 mg/kg/day up to 10 mg/kg/day. If AOP persisted after this treatment, oxygen therapy was escalated based on the infant’s condition. Preterm infants who did not meet the above criteria were categorized as responsive.

### Genetic analysis

For each preterm infant, 1 mL of umbilical artery blood was retained at birth, placed in EDTA anticoagulation tubes, and stored at -80 °C. DNA extraction was performed using the QIAamp DNA Blood Mini Kit (Qiagen, USA) according to the manufacturer’s instructions. DNA concentration was measured using a Ultraviolet-visible spectrophotometer (Thermo Fisher Scientific, USA). We selected four adenosine receptor genes (*ADORA1*, *ADORA2A*, *ADORA2B*, and *ADORA3*) and five dopamine receptor genes *(DRD1*, *DRD2*, *DRD3*, *DRD4*, and *DRD5*) for analysis. Tag-single-nucleotide polymorphisms (SNPs) specific to East Asian populations were screened using Halpview. A total of 22 SNPs were enrolled, including *ADORA1* (rs10920573 and rs6427994), *ADORA2A* (rs34923252 and rs2236624), *ADORA2B* (rs2015353), *ADORA3* (rs10776728, rs10857887 and rs1544224), *DRD1* (rs5326 and rs251937), *DRD2* (rs6278, rs6279, rs2283265, rs144999500 and rs1799978), *DRD3* (rs3732790, rs6762200, rs7625282, and rs6280), *DRD4* (rs936461 andrs3758653), and *DRD5* (rs77434921). Genotyping was performed using the MassArray Analyzer 4 system (Matrix-Assisted Laser Desorption Ionization time-of-flight mass spectrum, MALDI-TOF-MS). The primer information is provided in Supplementary Table 1.

### Statistical analysis

Statistical analysis was conducted using SPSS V25.0 and R V4.0.5. The Kolmogorov–Smirnov test was used to evaluate whether the variables conformed to a normal distribution. Normally distributed variables were presented as ‾*X* ± *S*, and independent-samples *t*-test was used for between-group comparisons. Abnormally distributed variables were presented as the median (interquartile range), and the Mann–Whitney *U*-test was used for their between-group comparisons. Categorical variables and genotypes were presented as rates (%), and the *χ2* test was used for their between-group comparisons. When conducting multiple comparisons, the Benjamini–Hochberg false discovery rate (FDR) was used for controlling the rate of false positives and obtain the *P*_*FDR*_. SNPs were analyzed using various models, including the recessive model (CC / CA *vs*. AA), dominant model (CC *vs*. AA / CA), log-additive model, over dominant model (CA *vs*. CC / AA), and codominant model (CC *vs*. CA *vs*. AA), wherein A represents the mutant allele and C represents the wild-type allele. When the values of Akaike’ information criterion (AIC) is the lowest, the genetic model is the optimal model for the SNP. Univariate logistic regression analysis was used to evaluate the association between SNPs and preterm infants’ response to caffeine citrate. Multivariable logistic regression was used to identify independent risk and protective factors and develop a nomogram to predict preterm infants’ response to caffeine citrate. Calibration and discrimination analyses and decision curve analysis were performed to evaluate nomogram performance. Both discrimination and calibration were assessed by bootstrapping with 1000 resamples. *P* < 0.05 was considered indicative of a statistically significant difference.

## Results

### General information

**Fig. 1:**
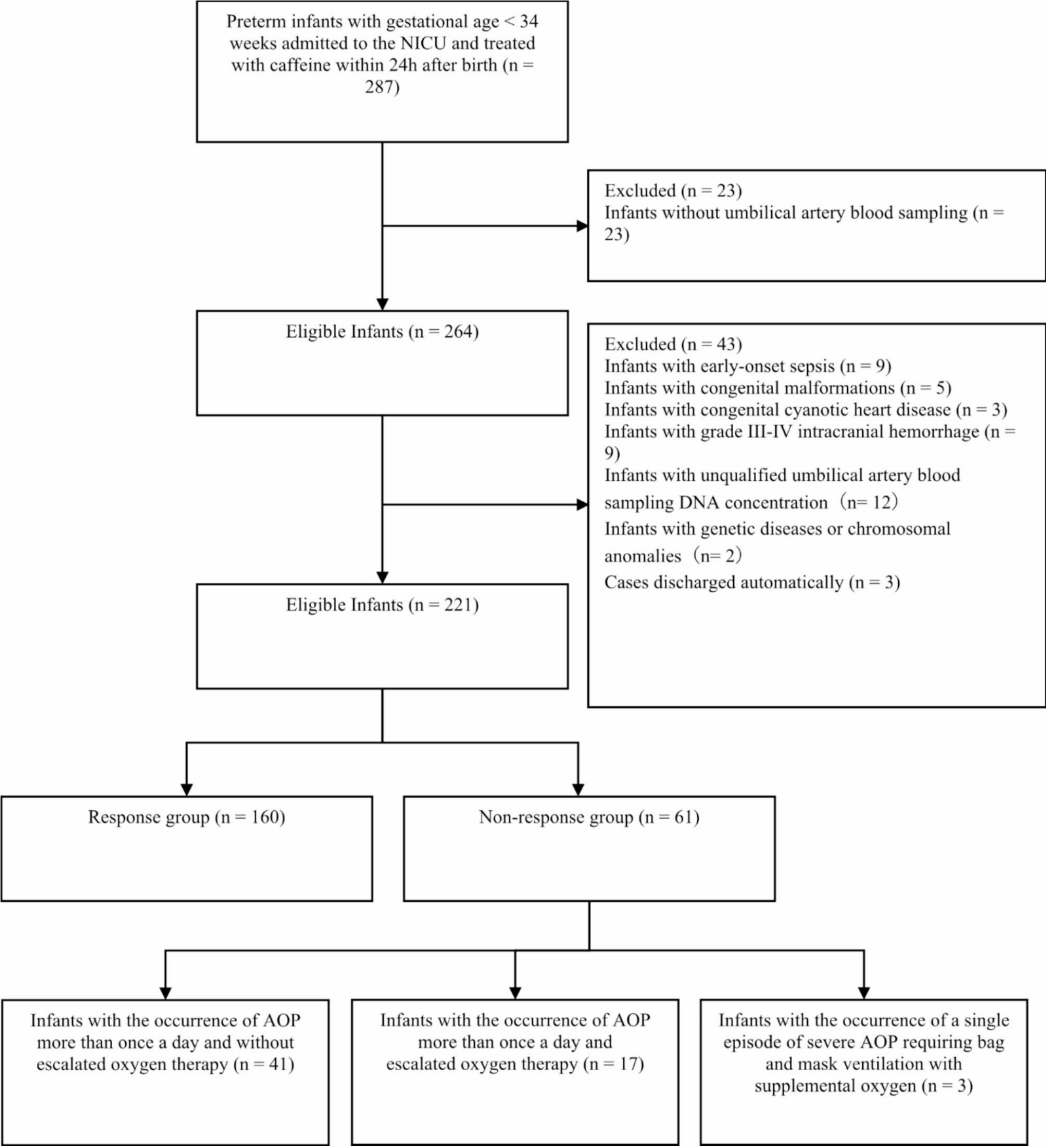
Flow diagram for preterm infants selection. AOP: apnea of prematurity

During the study period, 287 preterm infants with gestational age < 34 weeks were admitted to the NICU and treated with caffeine within 24 h after birth, among whom 66 did not meet the inclusion criteria (Fig. 1). Consequently, this study included 221 preterm infants with gestational ages ranging from 24 to 34 weeks and a median gestational age of 32.1 (30.29, 33.29) weeks. The birth weight of the preterm infants ranged from 650 g to 2570 g, with a median birth weight of 1650 g (1314–1920 g). Among them, 123 (55.7%) infants were male (Table 1).

**Table 1:** Comparison of baseline characteristics between the response group and non- response group

Variables	Total (*n* = 221)	Non-response group (*n* = 61)	Response group (*n* = 160)	Z/X^2^	*P*
Maternal age,‾*X* ± *S*, years	31.5 ± 4.7	31.5 ± 4.8	31.5 ± 4.7	-0.097	0.923
Gestational diabetes, *n* (%)	74 (33.5)	24 (39.3)	50 (31.4)	1.232	0.267
Gestational Hypertension, *n* (%)	33 (14.9)	9 (14.8)	24 (15.0)	0.002	0.963
Chorioamnionitis, *n* (%)	7 (3.2)	1 (1.6)	6 (3.8)	0.641	0.423
Premature rupture of membranes > 18 h, *n* (%)	55 (24.9)	19 (31.1)	36 (22.5)	1.767	0.184
Gestational age, *M* (*Q1*, *Q3*), weeks	32.1 (30.29, 33.29)	30.0 (28.2, 32.3)	31.04 (32.6, 33.4)	-5.446	**< 0.001**
Birth weight, *M* (*Q1*, *Q3*), grams	1650.0 (1314, 1920)	1313.0 (1016.5, 1652.5)	1742.5 (1479.3, 1965.0)	-5.454	**< 0.001**
Male, *n* (%)	123 (55.7)	32 (52.5)	91 (56.9)	0.349	0.555
Cesarean section, *n* (%)	151 (68.3)	45 (73.8)	106 (66.3)	1.154	0.283
SGA, *n* (%)	19 (8.6)	7 (11.5)	12 (7.5)	0.888	0.346
Completed antenatal steroids, *n* (%)	129 (58.4)	38 (62.3)	91 (56.9)	0.534	0.465
5 min Apgar score, *M* (*Q1*, *Q3*)	9 (9,10)	9 (8,9)	9 (9,10)	-3.388	**0.001**
NRDS, *n* (%)	64 (29.0)	26 (42.6)	38 (23.8)	7.646	**0.006**
PDA, *n* (%)	69 (31.2)	31 (50.8)	38 (23.8)	15.07	**< 0.001**

SGA: small for gestational age, NRDS: neonatal respiratory distress syndrome, PDA: patent ductus arteriosus

There were 61 preterm infants in the non-response group and 160 preterm infants in the response group. There was no statistically significant difference in maternal age, diabetes, hypertension or preeclampsia, chorioamnionitis, or premature rupture of membranes (> 18 h) between the two groups (*P* > 0.05). However, gestational age, birth weight and the 5-min Apgar score of the preterm infants were significantly lower in the non-response group than in the response group (*P* ≤ 0.001). In addition, the incidence of PDA and NRDS was significantly higher in the non-response group than in the response group (*P* < 0.001 and *P* = 0.006, respectively). There was no statistically significant difference in sex, mode of delivery, use of antenatal steroids, and SGA between the two groups (*P* > 0.05; Table 1).

### Treatment and clinical outcomes

Notably, the 221 preterm infants had a total oxygen supplementation time of 19 days (10.56–38.61 days) and underwent NIV for 13.43 days (6.43–29.43 days). In addition, 18.1% (40) of the preterm infants underwent IMV for a duration of 2.9 days (1.45–5.66 days). The time of invasive high-frequency mechanical ventilation was 2.0 days (0.1–3.6 days), and the rate of re-intubation after extubation was 2.3% (5). The mean maintenance dose of caffeine citrate was 5.13 mg/kg/day (5–5.46 mg/kg/day), and the median duration of caffeine citrate use was 15.5 days (9–28 days). The incidence rates of BPD (mild, moderate, and severe), NEC, ROP, and brain injury were 35.7% (79), 2.3% (5), 41.2% (91), and 14.9% (33), respectively. The mortality rate was 0.5% (1). The length of hospital stay was 30 days (21–47 days).

At initial admission, the non-response group had 9 preterm infants who did not undergo oxygen therapy, 39 underwent NIV, and 13 IMV. There were 20 premature infants who had escalated oxygen therapy due to AOP, including 5 who were transitioned from NIV to IMV. The response group had 20 preterm infants who did not undergo oxygen therapy, 118 underwent NIV, and 22 underwent IMV. There was no significant difference between the groups of admission without oxygen therapy, NIV, and IMV (*P* > 0.05).

Preterm infants in the non-response group had a longer total supplementation time (*P* < 0.001), longer NIV time (*P* < 0.001), longer IMV time (*P* = 0.039), higher rate of IMV (*P* < 0.01), and higher rate of re-intubation after extubation (*P* = 0.032). Preterm infants in the non-response group also had a higher average maintenance dose and longer duration of caffeine citrate use (*P* < 0.001). The incidence of BPD (*P* < 0.001), mild BPD (*P* < 0.001), moderate and severe BPD (*P* < 0.001), ROP (*P* < 0.001), and brain injury (*P* = 0.039) was higher in the non-response group, whereas the incidence of NEC and death did not significantly differ between the groups (*P* > 0.05). The length of hospital stay was also longer in the non-response group (*P* < 0.001; Table 2).

**Table 2:** Comparison of treatment information and complications between the response group and non-response group

Variables	Total (*n* = 221)	Non-response group (*n* = 61)	Response group (*n* = 160)	Z/X^2^	*P*
No oxygen therapy at admission, *n* (%)	29 (13.1)	9 (14.8)	20 (12.5)	0.197	0.657
Noninvasive mechanical ventilation at admission, *n* (%)	157 (71.0)	39 (63.9)	118 (73.8)	2.068	0.150
Invasive mechanical ventilation at admission *n* (%)	35 (15.8)	13 (21.3)	22 (13.8)	1.894	0.169
Total oxygen supplementation time, *M* (*Q1*, *Q3*), days	19.0 (10.6, 38.6)	39.5 (22.6, 52.8)	16.0 (8.9, 28.3)	-6.273	**< 0.001**
Noninvasive mechanical ventilation time, *M* (*Q1*, *Q3*), days	13.4 (6.4, 29.4)	30.3 (11.4, 44.5)	10.2 (5.1, 19.2)	-5.539	**< 0.001**
Invasive mechanical ventilation, *n* (%)	40 (18.1)	18 (11.3)	22 (36.1)	18.346	**< 0.001**
Invasive mechanical ventilation time, *M* (*Q1*, *Q3*), days	2.9 (1.5, 5.7)	3.5 (2.1, 7.6)	2.5 (0.9, 4.2)	-2.066	**0.039**
High-frequency ventilation mechanical time, *M* (*Q1*, *Q3*), days	1.8 (0.5, 4.0)	1.7 (0.7, 5.1)	2.0 (0.1, 3.6)	-1.043	0.297
Re-intubations after extubation, *n* (%)	5 (2.3)	4 (6.6)	1 (0.6)	4.602	**0.032** ^**a**^
Caffeine citrate average Maintenance dose, *M* (*Q1*, *Q3*), mg/kg/d	5.1 (5.0, 5.5)	5.7 (5.0, 9.8)	5.0^b^	-9.447	**< 0.001**
Caffeine citrate duration, *M* (*Q1*, *Q3*), days	15.5 (9.0, 28.0)	30.5 (17.0, 43.0)	12.5 (8, 21)	-6.147	**< 0.001**
BPD, *n* (%)	79 (35.7)	44 (72.1)	35 (21.9)	48.563	**< 0.001**
Mild BPD, *n* (%)	35 (15.8)	22 (36.1)	13 (8.13)	21.146	**< 0.001**
Moderate and severe BPD, *n* (%)	44 (19.9)	22 (36.1)	22 (13.8)	13.792	**< 0.001**
NEC, *n* (%)	5 (2.3)	1 (1.6)	4 (2.5)	0.000	1.000^a^
ROP, *n* (%)	91 (41.2)	40 (65.6)	51 (31.9)	20.705	**< 0.001**
Brain injury, *n* (%)	33 (14.9)	14 (23.0)	19 (11.9)	4.265	**0.039**
Death, *n* (%)	1 (0.5)	1 (1.6)	0	0.252	0.616^a^
Length of hospital stay, *M* (*Q1*, *Q3*), days	30.0 (21.0, 47.0)	50.5 (33.8, 65.0)	27 (20, 35)	-5.981	**< 0.001**

a: Continuity correction chi square test

b: The caffeine citrate average maintenance dose in the response group were 5.0 mg/kg/d

BPD: bronchopulmonary dysplasia, NEC: necrotizing enterocolitis, ROP: retinopathy of prematurity

### The association between caffeine citrate response and gene polymorphisms

Among the 22 SNPs, rs6279 and rs2283265 did not conform to the Hardy–Weinberg equilibrium, and rs144999500 did not detect mutations; therefore, these SNPs were excluded from further analysis, and 19 SNPs were finally included.

*ADORA1* rs10920573 (*P*_*FDR*_ = 0.049, recessive model), *ADORA2B* rs2015353 (*P*_*FDR*_ = 0.049, super-dominant model), *ADORA3* rs10776728 (*P*_*FDR*_ = 0.049, recessive model), *DRD3* rs7625282 (*P*_*FDR*_ = 0.049, super-dominant model), and *DRD3* rs6280 (*P*_*FDR*_ = 0.049, super-dominant model) were found to be associated with caffeine citrate response in preterm infants.

In the univariate logistic regression analysis, the CC genotype of *ADORA1* rs10920573 (TT / TC vs. CC: OR, 2.89; 95% CI, 1.30–6.40), TC genotype of *ADORA2B* rs2015353 (TT / CC vs. TC: OR, 2.56; 95% CI, 1.28–5.12), AG genotype of *DRD3* rs7625282 (AA / GG vs. AG: OR, 2.15; 95% CI, 1.17–3.96), and CT genotype of *DRD3* rs6280 (CC / TT vs. CT: OR, 2.36; 95% CI, 1.29–4.30) were identified as risk factors for non-response to caffeine citrate in preterm infants. Conversely, the AA genotype of *ADORA3* rs10776728 (TT / TA vs. AA: OR, 0.30; 95% CI: 0.11–0.80) was identified as a protective factor for caffeine citrate response in preterm infants (Table 3).

**Table 3:** Genotype distribution of adenosine and dopamine receptor gene polymorphisms among caffeine citrate response and non-response preterm infants

Gene	SNP	Genotype	Response group (*n* = 160)	Non-response group (*n* = 61)	OR (95CI%)	*P*	*P*_FDR_
*ADORA1*	rs10920573	T > C			**2.89 (1.30–6.40)**	**0.008** ^**a**^	**0.049**
		TT / TC	145	47			
		CC	15	14			
	rs6427994	A > C			1.91 (1.00-3.64)	0.047^a^	0.149
		AA / AC	17	68			
		CC	43	90			
*ADORA2A*	rs34923252	T > A			1.46 (0.64–3.33)	0.374^b^	0.474
		TT	51	141			
		AA / TA	10	19			
	rs2236624	T > C			2.15 (0.71–6.56)	0.168 ^b^	0.355
		TT	21	4			
		CC / CT	139	57			
*ADORA2B*	rs2015353	T > C			**2.56 (1.28**–**5.12)**	**0.007** ^**c**^	**0.049**
		TT / CC	123	40			
		CT	24	20			
*ADORA3*	rs10776728	T > A			**0.30 (0.11**–**0.80)**	**0.011** ^**a**^	**0.049**
		TT / TA	123	56			
		AA	37	5			
	rs10857887	A > G			0.69 (0.38–1.24)	0.209^a^	0.397
		AA / AG	71	33			
		GG	88	28			
	rs1544224	A > G			1.54 (0.85–2.80)	0.156^b^	0.355
		AA	86	26			
		GG / AG	73	34			
*DRD1*	rs5326	C > T			1.64 (0.61–4.38)	0.322^a^	0.474
		CC / CT	146	52			
		TT	12	7			
	rs251937	T > C			1.06 (0.59–1.91)	0.848^c^	0.848
		CC/ TT	81	30			
		TC	79	31			
*DRD2*	rs6279	G > C			1.24 (0.64–2.41)	0.521^b^	0.582
		GG	49	16			
		CC / GC	111	45			
	rs1799978	T > C			1.33 (0.32–5.48)	0.695^a^	0.734
		TT / TC	154	58			
		CC	6	3			
*DRD3*	rs6762200	C > T			1.92 (1.05–3.50)	0.033^c^	0.474
		CC / TT	105	31			
		CT	53	30			
	rs7625282	A > G			**2.15 (1.17**–**3.96)**	**0.013** ^**c**^	**0.049**
		AA / GG	114	33			
		AG	45	28			
	rs6280	C > T			**2.36 (1.29**–**4.30)**	**0.005** ^**c**^	**0.049**
		CC / TT	109	29			
		CT	51	32			
	rs3732790	T > A			0.59 (0.19–1.83)	0.357^a^	0.474
		TT / TA	143	57			
		AA	17	4			
*DRD4*	rs936461	A > G			1.28 (0.71–2.33)	0.414^a^	0.492
		AA / AG	83	28			
		GG	74	32			
	rs3758653	T > C			1.60 (0.87–2.95)	0.132^c^	0.355
		TT / CC	87	40			
		TC	73	21			
*DRD5*	rs77434921	G > A			0.65 (0.27–1.57)	0.333^b^	0.474
		GG	132	53			
		AA / GA	27	7			

a: recessive model b: dominant model c: over dominant model

SNP single nucleotide polymorphism, *ADORA1* adenosine A1 receptor gene, *ADOR2A* adenosine A2A receptor gene, *ADOR2B* adenosine A2B receptor gene, *ADORA3* adenosine A3 receptor gene, *DRD*1 dopamine D1 receptor gene, *DRD*2 dopamine D2 receptor gene, *DRD*3 dopamine D3 receptor gene, *DRD*4 dopamine D4 receptor gene, *DRD*5 dopamine D5 receptor gene

### Model for predicting caffeine citrate response

In the multiple logistic regression analysis adjusted for genotypes (*ADORA1* rs10920573, *ADORA2B* rs2015353, *ADORA3* rs10776728, *DRD3* rs7625282, and rs6280), gestational age, birth weight, Apgar score, prenatal hormones, NRDS and PDA, the CC genotype of *ADORA1* rs10920573 (aOR, 3.51; 95% CI, 1.34–9.25) and CT genotype of *DRD3* rs6280 (aOR, 3.19; 95% CI, 1.53–6.65) were identified as independent risk factors for non-response to caffeine citrate in preterm infants. Conversely, higher gestational age (aOR, 0.63; 95% CI, 0.53–0.75) was identified as an independent protective factor for caffeine citrate response in preterm infants (Table 4).

**Table 4:** Multivariable logistic regression analysis to identify independent risk and protective factors of non-response

Variables	B	*P*	aOR	95% CI
rs10920573	1.257	0.011	3.51	1.34–9.25
rs6280	1.159	0.002	3.19	1.53–6.65
Gestational age, weeks	-0.455	< 0.001	0.63	0.53–0.75

The multivariable logistic regression analysis was adjusted for genotypes (*ADORA1* rs10920573, *ADORA2B* rs2015353, *ADORA3* rs10776728, *DRD3* rs7625282 and rs6280), gestational age, birth weight, Apgar score, prenatal hormones, NRDS and PDA

**Fig. 2:**
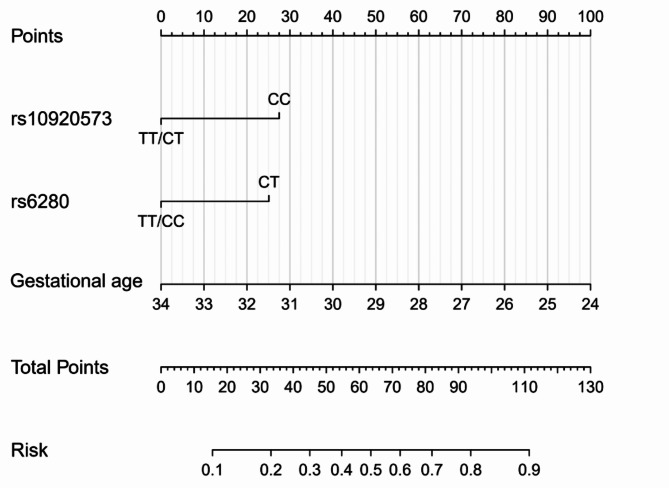
Nomogram for the prediction of caffeine citrate non-response in preterm infants

**Fig. 3:**
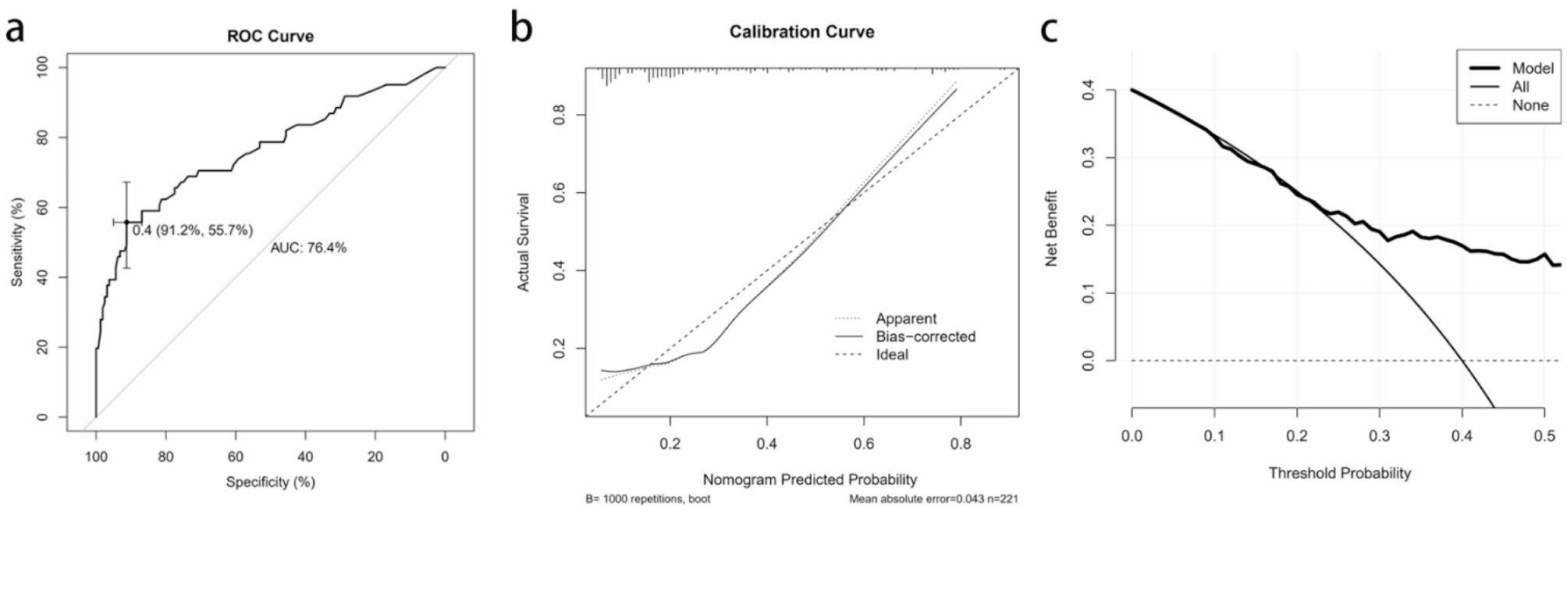
**a**: ROC curves. ROC receiver operating characteristic, AUC area under the ROC curve. **b**: Calibration curve for predicting probability of caffeine citrate non-response in preterm infants. **c**: Decision curve analysis in prediction of caffeine citrate non-response in preterm infants

We developed a nomogram to predict caffeine citrate response in preterm infants on the basis of multivariable logistic regression analyses, including the genotype of *ADORA1* rs10920573, the genotype of *DRD3* rs6280, and gestational age (Fig. 2). The area under the curve (AUC) of the receiver operating characteristic (ROC) curve was 0.764, and the concordance index was 0.764 (95% CI: 0.687–0.842; Fig. 3a). The calibration curve showed a high level of agreement between the predicted and actual probabilities, and this curve was close to the diagonal line (Fig. 3b). The clinical decision curve showed better net benefit in the predictive model (Fig. 3c).

## Discussion

In our study, besides adenosine receptor genes, we found for the first time that dopamine receptor gene polymorphisms are associated with caffeine citrate response in preterm infants. These new biomarkers may serve as predictors of caffeine citrate response in preterm infants. Furthermore, by combining the genotypes of adenosine receptor genes, dopamine receptor genes, and important clinical variables, we developed a nomogram for caffeine citrate response in preterm infants. This nomogram provided an intuitive prediction probability on the basis of genotypes and important clinical variables, offering reference for achieving personalized and precise caffeine citrate therapy.

In our study, 27.6% of preterm infants did not respond to standard-dose caffeine citrate treatment. Preterm infants in the non-response group had a lower gestational age, lower birth weight, and longer total duration of oxygen use, and these factors were associated with an increased incidence of ROP. Intermittent hypoxemia caused by AOP and prolonged mechanical ventilation are also important risk factors for the development of BPD and brain injury [18, 19]; we observed, indeed, a significantly higher incidence of BPD and brain injury in the non-response group. Therefore, it is particularly important to consider genetic factors to predict non-response to caffeine citrate treatment in preterm infants with AOP [20].

After adjustment for genotypes and clinical factors, multivariable logistic regression analysis revealed that the frequency of the *ADORA1* rs10920573 CC genotype was significantly increased in the non-response group than in the response group and that this frequency was an independent risk factor for non-response to caffeine citrate treatment in preterm infants. Adenosine receptors belong to the G protein-coupled receptor (GPCR) family, which includes inhibitory A1 and A3 receptors, as well as stimulatory A2A and A2B receptors. These receptors are direct targets for caffeine. A1 and A3 receptors inhibit the cyclic adenosine monophosphate (cAMP) signaling pathway via Gi/o proteins, and A2A and A2B ones stimulate this signaling pathway via Gs proteins [21]. It is generally believed that caffeine acts mainly by antagonizing A1 and A2A receptors at physiological concentrations [22]. Activation of A1 receptor induces sleep and inhibits respiration, activation of A2A receptor stimulates wakefulness and increases alertness [21]. While demonstrating contrasting effects at the cellular and analytical levels, the central arousal effects of caffeine are mediated through a combined action on A1 and A2A receptors. Inhibition of A1 receptors stimulates respiratory nerve output and inhibition of A2A receptors located on GABAergic neurons reduces inhibition of respiratory reflexes [23]. Therefore, mutations in A1 and A2A receptors may affect the effects of caffeine on respiration.

To our knowledge, this is the first study to focus on whether dopamine receptor gene polymorphisms affect the response to caffeine citrate treatment in preterm infants. We found that preterm infants with the *DRD3* rs7625282 AG genotype and rs6280 CT genotype showed non-response to caffeine citrate treatment. After adjusted for genotypes and clinical factors by multivariable logistic regression analysis, the rs6280 CT genotype still retained a significant association as an independent risk factor for non-response to caffeine citrate treatment in preterm infants. Dopamine receptors also belong to the GPCR family, which include stimulatory D1-like receptors (D1 and D5) and inhibitory D2-like receptors (D2, D3, and D4) [24]. Dopamine receptors and adenosine receptors have similar distribution in the brain and form functional heteromeric complexes co-expressed on cell membranes [25–27]. There is experimental evidence supporting the notion that dopamine receptors play a crucial role in the behavioral effects of caffeine in both animals and humans [28]. Dopamine receptor gene knockout or dopamine receptor blockade significantly reduces the stimulatory effects of caffeine on locomotion [28, 29]. Functional dopamine receptor gene polymorphisms can influence individual responses to caffeine [30]. Childs et al. [11] found that combinations of dopamine receptor gene polymorphisms with adenosine receptor gene polymorphisms lead to more variance in caffeine-induced anxiety than either SNP alone. In addition to interacting with adenosine receptors, the dopaminergic system is also involved in the regulation of respiration. Dopamine is present in almost all regions of the medullary respiratory center, and dopamine receptors are also expressed in the respiratory tract [31, 32]. Animal studies have confirmed that when knocking out the nuclear receptor transcription factors associated with dopamine neuron development in mice, the mice developed frequent apnea postnatally and failed to establish normal ventilation [33]. *DRD3* rs6280 mutation is a missense mutation involving the substitution of serine for glycine at the 9th position in the N-terminal domain. D3 receptors are densely expressed in the limbic subcortical region and the striatum, and form A2A-D3 heteromeric complexes with A2A receptors at the nerve cell membrane [27, 34]. Activation of D3 receptor inhibits cAMP activity, whereas activation of A2A receptor stimulates cAMP activity. Therefore, D3 receptor and A2A receptor antagonize each other at the second messenger level. Studies have shown that the rs6280 homozygous genotype leads to a 4–5-fold increase in D3 receptor affinity and an increase in the density of D3 receptors in the brain [35]. Accordingly, we hypothesized that preterm infants with the CT genotype exhibit lower activity levels of D3 receptors, resulting in reduced inhibition of the A2A receptor signal and an attenuated caffeine effect. However, relevant studies supporting that the rs6280 polymorphism directly affects respiratory regulation and subsequently leads to apnea are currently lacking, and thus, further studies are needed to clarify the specific mechanism.

Nowadays, a growing number of drug–gene interactions are demonstrated to have clinical validity [36]. As an emerging component of precision medicine, gene-guided dosing strategies can improve the efficacy and accuracy of existing drugs [37]. In our study, corrected by multivariable logistic regression analysis, we combined genetic factors and important clinical variables to develop a nomogram. This model may provide an early prediction of the response to caffeine citrate in preterm infants, which could help achieve individualized caffeine citrate treatment. However, implementing our model in clinical practice still poses challenges. On one hand, we have not incorporated epigenetic factors. As a crucial component of precision medicine, epigenetic modifications may vary with different clinical variables and genotypes in preterm infants, thereby affecting the risk of disease development and drug response [38–41]. On the other hand, currently, fetus and infant genotyping during pregnancy or at birth is not routinely performed. Nevertheless, the next generation sequencing (NGS) technology and molecular analyses, employing large-scale parallel sequencing strategies, are profoundly altering the landscape of clinical genomics. Not only can comprehensive genetic information be rapidly and accurately obtained, but rare genetic variations can also be unveiled [42, 43]. These strategies facilitate the diagnosis of complex and rare diseases, as well as the identification of individualized responses to drugs [44, 45]. We made an attempt and to some extent provided some evidence for future individualized caffeine treatment in premature infants. Further studies might be required to validate the current model and integrate the predictive model with the potent tool of NGS to further elucidate the pharmacogenetic variations associated with caffeine citrate.

Actually, there are several limitations to our study. First, the subjects of this study were limited to a single-center population in China, the results can be different and not confirmed in other ethnicities or populations. Second, the non-response group had a lower gestational age, which might itself explain the presence of AOP despite caffeine citrate treatment. Larger sample size and more homogeneous cohorts will be needed to confirm that gene polymorphism can alone cause a poor response to the caffeine citrate. Third, some infants who used caffeine citrate to prevent AOP may not have actually developed apnea and were classified in the response group, which may have limited the accuracy of the results. Besides, the employment of a panel of a priori established genes, rather than the entire exome, might overlook other potential genomic anomalies or polymorphisms that contribute to a diminished response to treatment and the progression of the disease. The impact of factors associated with caffeine metabolism were not take into account as well, and then our predictive model may not be comprehensive. Finally, the use of umbilial cord blood might be not the best optimal for genetic analysis and the validation at in vitro levels was lacked in our study. Additional studies with larger samples are needed to verify the precise contribution of gene polymorphisms to treatment of AOP.

## Conclusions

In conclusion, besides adenosine receptor gene polymorphisms, dopamine receptor gene polymorphisms also played a role in the response of preterm infants with AOP to caffeine citrate treatment, and may be another biomarker for predicting caffeine citrate response in preterm infants. Polymorphisms in *ADORA1* and *DRD3* genes and gestational age were independently associated with caffeine citrate response. We combined *ADORA1* and *DRD3* genes polymorphisms as well as gestational age to develop a nomogram for predicting caffeine citrate response in preterm infants. Further research may require integration with relevant analyses of NGS, which can help to obtain more genetic information and avoid inappropriate treatments in categories with peculiar genomic profiles, thus providing a more comprehensive reference for the personalized medication of caffeine citrate in premature infants and the precision treatment of AOP.

## Electronic Supplementary Material

Below is the link to the electronic supplementary material.

Supplementary Material 1

## Data Availability

The datasets generated and/or analysed during the current study are not publicly available but are available from the corresponding author on reasonable request. Due to the data were used under license for the current study, and so are not publicly available.

## Abbreviations

AOP: Apnea of prematurity
PDA: Patent ductus arteriosus
BPD: Bronchopulmonary dysplasia
NRDS: Neonatal respiratory distress syndrome
ROP: Retinopathy of prematurity
SGA: Small for gestational age
NEC: Necrotizing enterocolitis
ROC: Receiver operating characteristic
