# Supplementary material for: The association of gene polymorphisms of adenosine and dopamine receptors with the response to caffeine citrate treatment in infants with apnea of prematurity: a prospective nested case-control study
Source: Ital J Pediatr. 2024 Oct 29;50:225. doi: 10.1186/s13052-024-01776-w (PMC11520374; doi:10.1186/s13052-024-01776-w)
Supplement: Supplementary file 1 — Supplementary Material 1 [file 13052_2024_1776_MOESM1_ESM.docx]

**Supplementary Table 1** The primer information of genes

| Gene | SNP | Forward primer (5' to 3') | Reveres primer (5' to 3') | Extension primer |
| --- | --- | --- | --- | --- |
| *ADORA1* | rs10920573 | ACGTTGGATGCCTGATTCTTCCCATTTTCG | ACGTTGGATGCTCTTACTGCATCCTCACTC | gggttGTGACCTTGCTTCCCA |
|  | rs6427994 | ACGTTGGATGTGCCTGACCATCCCATGAG | ACGTTGGATGTGGCTCCTCTGCAGTCTCA | aggaaCATCCCATGAGCAGTCCAG |
| *ADORA2A* | rs34923252 | ACGTTGGATGTGAGAATGGCGTCTGAGTTC | ACGTTGGATGCTCATACACAACACATGCC | cctcgTGGCGTCTGAGTTCGTTTCCTAC |
| *ADORA2B* | rs2015353 | ACGTTGGATGTTCCTGCATGAAAGCAGCAC | ACGTTGGATGCCTTTCAGGCAGAAGCTAAA | GCACATGTTGTTCTC |
| *ADORA3* | rs10776728 | ACGTTGGATGTTCTGGGAACCAGACACTTG | ACGTTGGATGATAATCCCTTTGGATGGTGC | ttgcACCAAGTGGGTCCCCAAATAAC |
|  | rs10857887 | ACGTTGGATGGCGTTCTGGTGTTTACTAAC | ACGTTGGATGCAACTAACAAGAGGACCCTG | ccttgTTACTAACAAACACTTC |
| *DRD1* | rs5326 | ACGTTGGATGCAGTCAGATTTCCAGGAGTC | ACGTTGGATGTCATGGAATGTTGGTGAGGC | agggaTGTGCAAAGTGCTGCCT |
|  | rs251937 | ACGTTGGATGAAGGCAACATGTGCCATAC | ACGTTGGATGCAAGGAAGAAAACACAGGC | cccTACATAACTGAAGAAACTTCCA |
| *DRD2* | rs6278 | ACGTTGGATGATACATTGCAGGGCCGTCAG | ACGTTGGATGAAGAGGAGCCCTCATCTTG | ggtgGCAGGGCCGTCAGAAGGCAG |
|  | rs6279 | ACGTTGGATGGTGTGAACTGTCCATCTCTC | ACGTTGGATGTAGGGTTGCTGGAGCCTGAG | agggaTGTGCAAAGTGCTGCCT |
|  | rs2283265 | ACGTTGGATGATGAGGAAACAGGCTCATAG | ACGTTGGATGCTAGACGCATCAGGTTCAG | aagtCTCATAGAAGGTAAG |
|  | rs144999500 | ACGTTGGATGTCATGGTCTTGAGGGAGGTC | ACGTTGGATGAAAGACCACCCCAAGATTGC | TTGAGATCCAGACCATGC |
|  | rs1799732 | ACGTTGGATGAAAGGAGCTGTACCTCCTCG | ACGTTGGATGCTCAAAACAAGGGATGGCG | CCCTCCTACCCGTTC |
|  | rs1799978 | ACGTTGGATGCTTGTTTTGAGGCGGGAAC | ACGTTGGATGAGGACCCAGCCTGCAATCA | cccCCTGCAATCACAGCTT |
| *DRD3* | rs3732790 | ACGTTGGATGGTGTAATGAATCATGCCTC | ACGTTGGATGGGGAGCTTCCAAGTGATAAG | atgcATCATGCCTCTGATGACAATTT |
|  | rs6762200 | ACGTTGGATGGAGAGTTAATAACTATTGG | ACGTTGGATGGGCCTTCTAATCAATTGTTAC | ccccgTTCTTTTTATCTCATTCAG |
|  | rs7625282 | ACGTTGGATGTCATTGCAGAGGAAGAATC | ACGTTGGATGTCTGTCTCCCTGTACTAGC | TGACCTTGGACAAAG |
|  | rs6280 | ACGTTGGATGCTGGCACCTGTGGAGTTCT | ACGTTGGATGTCTGGGCTATGGCATCTCTG | tcgtCCCCACAGGTGTAGTTCAGGTGGC |
| *DRD4* | rs936461 | ACGTTGGATGCTGTCGTGTCTTTCTCCTG | ACGTTGGATGCCCCTCTGACCCTGGGCA | ccgGGCCTCCTCGCGAGCCGAACCT |
|  | rs3758653 | ACGTTGGATGGAAAATACCTCTCAGGTCAC | ACGTTGGATGGAGAAAGTGCTTGCAAAGCG | actcGCAAAGCGCAGCAGAGA |
| *DRD5* | rs1967551 | ACGTTGGATGCTCATGGATCTGCATAACCG | ACGTTGGATGCATGATAAAGGGAGCAGCAC | ggaTGTGTGCGTGCTTGTCA |
|  | rs77434921 | ACGTTGGATGATGTTGGTCCAGGGTACAG | ACGTTGGATGGCATGGTGAGGTCTAGAAAC | ccTCCTAGTCTCCCCCT |
